# Manual of Chemistry

**Published:** 1895-06

**Authors:** 


MANUAL OF CHEMISTRY.—A Guide to Lectures and Laboratory work for Beginners in Chemistry. A text-book specially adapted for students of Medicine, Pharmacy, and Dentistry. By W. Simon, Ph. D., M. D., Professor of Chemistry in College of Physicians and Surgeons, etc., etc., Baltimore. Fifth edition, thoroughly revised with forty-four illustrations and eight colored plates representing sixty-four chemical reactions. Publishers: Lea Brothers & Co., Philadelphia, 1895.

The steadily increasing demand for this valuable and comprehensive manual of chemistry stimulated the author to prepare this new edition, and the manner in which he has performed such a duty is highly commendable, and the result is a work which is worthy of all praise for its completeness and excellence. The old classification of metals and non-metals, organic and inorganic compounds has been retained, for the reason that it is well adapted to the instruction of beginners in chemistry.

This work is also a most useful one to students of medicine, dentistry and pharmacy, for the reason that it furnishes an admirable selection of material bearing upon the laws and phenomena of chemistry under seven main divisions, treating respectively of the fundamental properties of matter, the principles of the science, the non-metallic and the metallic elements and their compounds, analytical, organic and physiological chemistry. Every one interested in chemistry will appreciate the value of the colored plates of reactions, which give a permanent and accurate series of standards for comparison of tests.

In medical and dental practice, important pathological and toxicological questions depending on the text-tube may with certainty be referred to this series of colors and color-changes.

